# Predicting active enhancers with DNA methylation and histone modification

**DOI:** 10.1186/s12859-023-05547-y

**Published:** 2023-11-02

**Authors:** Ximei Luo, Qun Li, Yifan Tang, Yan Liu, Quan Zou, Jie Zheng, Ying Zhang, Lei Xu

**Affiliations:** 1 Institute of Fundamental and Frontier Sciences, University of Electronic Science and Technology of China, Chengdu, Sichuan China; 2 School of Electronic and Communication Engineering, Shenzhen Polytechnic University, Shenzhen, Guangdong China; 3 Department of Pain, The Affiliated Traditional Chinese Medicine Hospital of Southwest Medical University, Luzhou, Sichuan China; 4 Department of Anesthesiology, The Affiliated Traditional Chinese Medicine Hospital of Southwest Medical University, Luzhou, Sichuan China; 5 Yangtze Delta Region Institute (Quzhou), University of Electronic Science and Technology of China, Quzhou, Zhejiang China

**Keywords:** Enhancer RNAs, CAGE-seq, H3K27ac, DNA methylation

## Abstract

**Background:**

Enhancers play a crucial role in gene regulation, and some active enhancers produce noncoding RNAs known as enhancer RNAs (eRNAs) bi-directionally. The most commonly used method for detecting eRNAs is CAGE-seq, but the instability of eRNAs in vivo leads to data noise in sequencing results. Unfortunately, there is currently a lack of research focused on the noise inherent in CAGE-seq data, and few approaches have been developed for predicting eRNAs. Bridging this gap and developing widely applicable eRNA prediction models is of utmost importance.

**Results:**

In this study, we proposed a method to reduce false positives in the identification of eRNAs by adjusting the statistical distribution of expression levels. We also developed eRNA prediction models using joint gene expressions, DNA methylation, and histone modification. These models achieved impressive performance with an AUC value of approximately 0.95 for intra-cell prediction and 0.9 for cross-cell prediction.

**Conclusions:**

Our method effectively attenuates the noise generated by stochastic RNA production, resulting in more accurate detection of eRNAs. Furthermore, our eRNA prediction model exhibited significant accuracy in both intra-cell and cross-cell validation, highlighting its robustness and potential application in various cellular contexts.

**Supplementary Information:**

The online version contains supplementary material available at 10.1186/s12859-023-05547-y.

## Background

Enhancers play a critical role in controlling gene transcription by interacting with cis-acting DNA regulatory regions. Unlike proximal regulatory elements, enhancers are located at distal positions from target genes [1–4]. Over long genomic distances, it can approach distant promoters and enhance the expression of its target genes. It exhibits dynamic characteristics that vary across different tissues and lineages. Identifying enhancers can be challenging due to their diverse properties in different tissues and their ability to act bidirectionally with respect to their target genes. With the advent of high-throughput sequencing technology, researchers have discovered that active enhancers are capable of transcribing DNA into RNA, known as enhancer RNAs (eRNA) [5, 6]. Unlike the mRNAs produced by promoters, eRNAs are typically short, 5-capped, transcribed bidirectionally in the nucleus, abundant, no polyadenylated, and relatively unstable [7, 8]. They serve as a hallmark of enhancer activity and can interact with proteins to regulate gene expression. The expression level of eRNAs is positively correlated with the expression of their target genes, and knocking down eRNAs leads to decreased expression of the corresponding target genes [6, 9, 10]. Furthermore, searchers have found that the eRNAs are tissue-specific and can provide explanatory power for some cancer phenotypes [8, 11, 12].

Regarding the database of enhancers, in the FANTOM5 enhancer atlas, 65,339 candidate eRNA enhancers were identified by detecting eRNA expression through cap analysis of gene expression (CAGE) sequencing across 1829 cell lines, providing coverage of enhancers in the majority of human cell types and tissues [13–15]. In addition, a comprehensive database, EnhancerDB, has been proposed [16]. It integrates experimental data from the FANTOM5 project with valuable informations on transcription factors and microRNAs that interact with enhancers. By leveraging these reliable resources, EnhancerDB and FANTOM5 provide more comprehensive and accurate tools for the identification of enhancers.

Recently, a plethora of computational methods have emerged for the identification of enhancers. As Table 1 shows, these methods can be divided into two categories: unsupervised learning and supervised learning [17–27]. In unsupervised learning methods, the main goal is to identify the histone modification patterns and the regulatory elements. In supervised learning. It is mainly based on two categories algorithm models: SVM and deep learning. The input features include DNA sequence information and histone modifications. For DNA sequence information, it is important to note that the positions of enhancers can vary across different states of the same cell, which means that relying solely on fixed features may limit the ability to identify enhancer sites accurately. The advancement of computational methods has significantly expanded the repertoire of predicted enhancers across various cell lines. However, there are only two reports on the prediction of eRNA. Zhu et al. performed logistic regression on eRNA based on histone modified signals. And Zhang et al. performed a deep learning framework for identifying tissue-specific eRNAs. But neither of these methods pays attention to the noise problem in CAGE-seq data. Additionally, there are limitations on availability.

**Table 1:** Published prediction methods for enhancer and eRAN

Method name	Target	Features	Algorithm model	Years
ChromHMM	Chromatin state	Histone modifications + TF binding	HMM	2012
ChroModule	Chromatin state	Histone modifications + open chromatin	HMM	2013
Segway	Regulatory pattern	Histone modifications + TF binding + open chromatin	DBN	2013
ChromeGenSVM	Enhancer	Histone modifications	SVM	2012
RFECS	Chromatin state	Histone modifications	RF	2013
Enhancer-CRNN	Enhancer	Histone modifications	RNN	2019
kmer-SVM	Regulatory pattern	DNA sequence	SVM	2013
iEnhancer-2L	Enhancer/strength	DNA sequence	SVM	2016
SeqEnhDL	Enhancer	DNA sequence	MLP, CNN, and RNN	2021
iEnhancer-RD	Enhancer/strength	DNA sequence	DNN	2021
LSTMAtt	Enhancer/strength	DNA sequence	Bi-LSTM	2022
Logistic Regression Model (Without name)	eRNA	Histone modifications	Logistic regression	2013
DeepITEH	Tissue-specific eRNAs	DNA sequence + histone modifications	Bert + Bi-LSTM + DNN	2023

In fact, previous studies analyzing CAGE-seq data have struggled to effectively account for the inherent stochasticity in RNA production, resulting in noisy measurements of eRNA expression.

In this study, we have introduced novel statistical methods and prediction models that effectively filter out the noise associated with eRNA expression. Using the FANTOM5 database, we first fitted the data distribution of eRNA expression obtained from CAGE-seq using maximum likelihood estimation to identify highly reliable and effective eRNA expression. Subsequently, we developed a user-friendly eRNA prediction model that exhibits reduced reliance on specific omics data, thereby enabling researchers to predict the eRNA with greater accuracy.

## Results

### Effectively remove the noise of eRNA

**Fig. 1:**
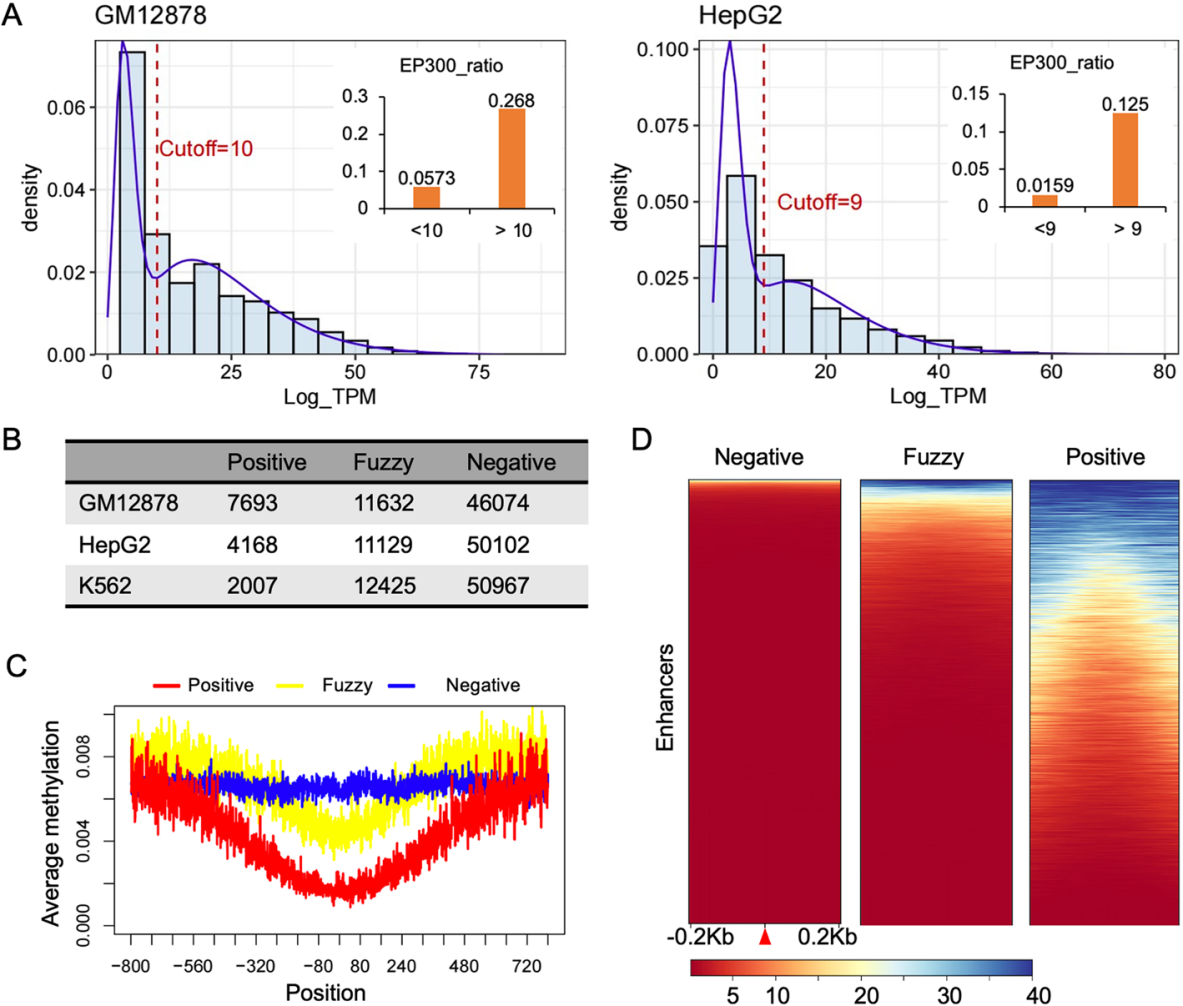
The distribution of eRNA expression and the labeled samples used for model training and evaluation. **A** The distribution of eRNA expression. By fitting the data distribution, false-positives in eRNA recognition can be reduced. **B** The number of samples labeled in GM12878, HepG2, and K562 cells. **C** There were significant differences in the expression of DNA methylation levels among the three types of samples. **D** H3K27ac enrichment in three types of samples in GM12878

eRNAs are expressed at relatively low levels, so using the CAGE-seq method to detecte eRNA is susceptible to random transcriptional noise [28]. In FANTOM 5 database, the eRNA expression level is quantified by transcript per million (TPM). Previous studies have not extensively examined the noise introduced by CAGE-seq, often considering all measured RNA as eRNA expression. However, in this study, we observed that the data distribution of expression values obtained through CAGE-seq might comprise two distinct distributions. Figure 1A shows the distribution of eRNA expression, indicating the presence of different data distribution within the measured expression data. This observation suggests the existence of potential noise or variations in the measured eRNA expression values, which necessitates further investigation and analysis.

In previous studies, the determination of eRNA production by enhancers was often based on a threshold, typically considering TPM values greater than 0 as indicative of eRNA expression. However, in this study, we took a different approach by fitting the data distribution and estimating its parameters. This allowed us to capture both the distribution of noise signals and the true expression of eRNA more accurately. Using this method, we identified a total of 11,584 and 6939 true eRNAs in GM12878 and HepG2 cells. By employing this approach, we were able to reduce false positives in eRNA identification, improving the reliability of our results. EP300, an important marker of active enhancers, was used in our analysis. We separately calculated the overlap ratio of EP300 with the regions identified as noisy and true eRNA, based on the designated cutoff. In GM12878, we observed overlap ratios of 0.0573 and 0.268 for EP300 with the regions identified as noise and true eRNA, respectively. Similarly, in HepG2, the overlap ratios were 0.0159 and 0.125 for EP300 with noise regions and true eRNA regions. These overlap ratios indicate a significant difference between the two categories. By fitting the data distribution and estimating its parameters, we were able to preliminarily eliminate the noise associated with eRNA.

It is crucial to get more accurate and reliable samples when building an accurate model. To enhance the accuracy of the model, we relied on additional omics data profiles to obtain more precise eRNA information. In this study, we utilized DNase hypersensitivity sites as indicators of chromatin states. Enhancers that have the ability to generate eRNA are generally located in open chromatin regions. By incorporating DNase-chip peak data, we established a more stringent approach for defining candidate regions. This allowed us to identify more accurate positive and negative samples. Figure 1B illustrated the distribution of positive samples in GM12878, HepG2, and K562 cells, with 7693, 4168, and 2007 positive examples defined, respectively. Additionally, a total of 46,074, 50,102, and 50,967 negative examples were also defined separately. By employing such a refined strategy, we aimed to improve the accuracy and reliability of our model by utilizing more precise and better-defined positive and negative samples.

Next, the DNA methylation level and the distribution of H3K27ac for the three groups of samples were calculated. As Fig. 1C, D show, the DNA methylation level and the distribution of H3K27ac were different between negative and positive samples in GM12878. Positive samples exhibited the expected absence of DNA methylation. In contrast, negative samples displayed higher levels of DNA methylation, particularly in proximity to the enhancer center regions. This differential DNA methylation provides evidence to support our initial biological hypothesis regarding the association between eRNA production and DNA methylation. Regarding the distribution of H3K27ac, negative samples exhibited a lack of H3K27ac signal, as expected. However, positive samples showed higher levels of H3K27ac compared to fuzzy regions. This enrichment of H3K27ac in positive samples further validates our hypothesis that enhancers producing eRNA events are often associated with increased levels of H3K27ac. Taken together, the distinctive patterns in DNA methylation and H3K27ac distribution observed between negative and positive samples in GM12878 cells provide strong evidence supporting our initial biological hypothesis, reaffirming the relationship between eRNA production, DNA methylation, and the enrichment of H3K27ac in the central region of enhancers.

### Intra-cell validation

**Fig. 2:**
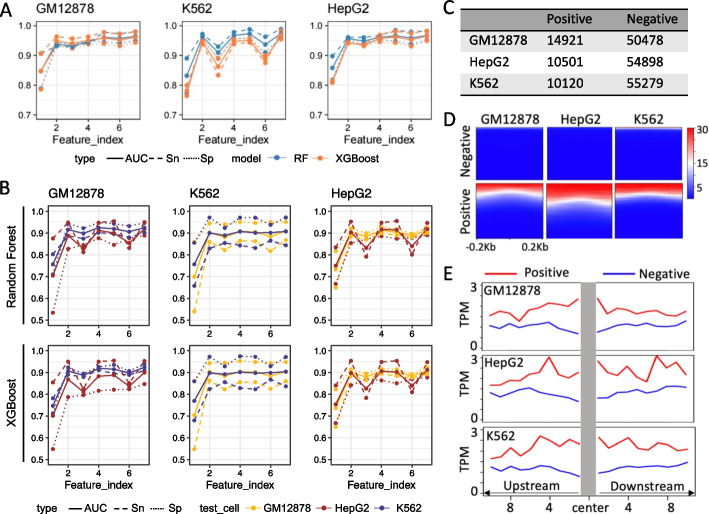
Performance of the model. **A** The performance of the model trained and tested in the same cell. **B** The performance of the model was tested in cross-cells. **C** The model used identified eRNA in three cells. The counts of eRNA and negative regions were identified in three cells. **D** The heatmaps of H3K27ac signal in different regions. **E** The average gene expression value within the enhancer regions was calculated

The Random Forest (RF) and Extreme Gradient Boosting (XGBoost) are high-performing machine-learning algorithms and can better perform imbalanced multiclassification. We employed algorithms to predict eRNA. They were evaluated on the independent test set using different combinations of omics features in the same cell. As shown in Fig. 2A, when using DNA methylation as a single feature, the model exhibited a high sensitivity (Sn) in predicting eRNA. However, the specificity (Sp) was lower than the sensitivity. This could be attributed to the presence of open chromatin regions with low levels of DNA methylation that do not actually transcribe eRNA. These findings suggest that additional necessary elements might be required for the transcription of eRNA when chromatin is open. Solely relying on DNA methylation data may lead to erroneous predictions of regions that do not actually produce eRNA.

To improve the accuracy of the model, histone modification signals were incorporated as additional features. When both H3K27ac and DNA methylation were used as features, the AUC values of RF in GM12878, K562, and HepG2 were 0.9374, 0.9630 and 0.9564, respectively. The AUC values of XGBoost model were consistently higher than 0.943 across all three cell types. It was observed that using H3K27ac as a feature yielded better performance compared to using H3K9ac. Furthermore, the inclusion of gene expression as a feature significantly improved the predictive capabilities of the model. The combination of gene expression, DNA methylation, and H3K27ac as features led to a substantial increase in the predicted AUC of the RF model, exceeding 0.961. These results highlight the robustness of using omics data, such as H3K27ac and DNA methylation, for accurate prediction of eRNA.

### Validation of enhancer predictions cross-cell type

To evaluate the performance of our supervised method across various cell types, we conducted cross-cell validation. The training data and testing data were extracted from different cell types. In three cells, 539 positive regions and 34,401 negative regions were shared (as shown in Additional file 1). Shared negative regions account for 74.66% of all negative regions, while the shared proportion of positive cases is only 4.75%. We assessed the performance of the models using different combinations of features, including DNA methylation and histone modification signals like H3K9ac and H3K27ac. The results demonstrated that models incorporating both DNA methylation and H3K27ac features achieved high AUC values, indicating their effectiveness in predicting eRNA expression across different cell types (as shown in Fig. 2B). Notably, H3K27ac exhibited superior performance compared to H3K9ac in terms of prediction accuracy and suitability as a feature. Furthermore, we observed that the random forest model outperformed the XGBoost model in our experiments. This suggests that the random forest algorithm was better suited for accurately predicting eRNA expression levels in different cell types when utilizing DNA methylation and H3K27ac as features.

### Comparison with other methods

We focus on predicting eRNA, which is nascent RNAs transcribed from active enhancers When compared to two published eRNA methods, the regression model cannot be directly compared due to the inability to obtain valid code, and the DeepITEH model depends on more histone modifications and complex DNA sequence feature extraction methods with input feature limitations. We have compared our method with the performance of other DNA-based enhancer activity prediction algorithms. Additionally, we compared and analyzed our prediction results with the ChromHMM annotation results that annotates strong and weak enhancers. When compared with two recently published methods (iEnhancer-RD and LSTMAtt), we found that only using DNA sequences is not sufficient for eRNA prediction. As shown in Table 2, iEnhancer-RD and LSTMAtt display very low sensitivity, MCC, and F1-score. iEnhancer-RD and LSTMAtt display very low sensitivity, MCC, and F1-score. Our method, which relies on DNA methylation and H3K27ac as input features and utilizes both RF and XGBoost models, outperforms iEnhancer-RD and LSTMAtt. eRNA undergoes changes across different cells and tissues, while DNA sequences remain unchanged. Therefore, predicting eRNA requires introducing other dynamic signals. Our method depends on WGBS and H3K27ac, which are relatively easy to obtain and have low sequencing costs. Additionally, the traditional algorithms we used have low computational requirements, indicating high feasibility of our method.

**Table 2:** Comparison with two recently published methods, iEnhancer-RD and LSTMAtt

Cell	Method	Sn	Sp	AUC	MCC	F1-score
GM12878	iEnhancer-RD	0.1323	0.9471	0.6494	0.1135	0.1774
	LSTMAtt	0.2838	0.8068	0.2290	0.0783	0.2325
	eRNA_RF_methyl_H3K27ac	0.9315	0.9433	0.9374	0.7942	0.8203
	eRNA_RF_methyl_H3K27ac_gene	0.9780	0.9445	0.9613	0.8277	0.8467
	eRNA_XGBoost_methyl_H3K27ac	0.9634	0.9317	0.9475	0.7890	0.8122
	eRNA_XGBoost_methyl_H3K27ac_gene	0.9780	0.9397	0.9589	0.8166	0.8363
K562	iEnhancer-RD	0.1361	0.9855	0.7181	0.1690	0.1800
	LSTMAtt	0.3849	0.8132	0.3131	0.0956	0.1257
	eRNA_RF_methyl_H3K27ac	0.9719	0.9541	0.9630	0.6491	0.6207
	eRNA_RF_methyl_H3K27ac_gene	0.9883	0.9575	0.9729	0.6721	0.6444
	eRNA_XGBoost_methyl_H3K27ac	0.9386	0.9536	0.9461	0.6271	0.6020
	eRNA_XGBoost_methyl_H3K27ac_gene	0.9442	0.9573	0.9508	0.6465	0.6239
HepG2	iEnhancer-RD	0.1752	0.9709	0.7260	0.2001	0.2276
	LSTMAtt	0.3667	0.8213	0.3012	0.1268	0.2087
	eRNA_RF_methyl_H3K27ac	0.9612	0.9515	0.9564	0.7520	0.7559
	eRNA_RF_methyl_H3K27ac_gene	0.9849	0.9476	0.9663	0.7535	0.7534
	eRNA_XGBoost_methyl_H3K27ac	0.9410	0.9453	0.9431	0.7191	0.7242
	eRNA_XGBoost_methyl_H3K27ac_gene	0.9812	0.9429	0.9620	0.7360	0.7351

### Prediction of transcribed eRNA in three cell lines

Utilizing the model trained within each individual cell type, we were able to predict eRNA regions across all three cell types, including those regions that were previously defined as fuzzy. This approach, which involved leveraging accurate data for model training and subsequently utilizing the trained model to predict fuzzy regions, effectively improved the identification of eRNA regions. By employing this strategy, we were able to achieve higher accuracy compared to solely relying on the variability of eRNA expression. The RF model, utilizing joint DNA methylation and H3K27ac as features, yielded high values for AUC, Sn, and Sp. This model, being characterized by these features, proved to be an effective tool for analyzing and identifying eRNA regions in all three cell lines.

As Fig. 2C shows, 14,921, 10,501, and 10,120 eRNAs were identified in GM12878, HepG2, and K562, respectively. To assess the overlap between the identified eRNA regions and EP300, the overlap ratios were calculated, resulting in ratios of 0.2486, 0.1032, and 0.3328 for the three cell lines, respectively. In contrast, the ratios for regions defined as negative samples were much lower, with values of 0.0086, 0.0011, and 0.0138. The presence of H3K27ac signal was found to be associated with eRNA and reflected enhancer activity. The heatmaps of the H3K27ac signal in the identified eRNA and negative regions are shown in Fig. 2D. Notably, there was a significant enrichment of H3K27ac signal upstream and downstream of the identified eRNA regions. In addition, the mean values of 10 genes located upstream and downstream of the enhancer regions were calculated. The gene expression levels were measured by TPM. As shown in Fig. 2E, the gene expression levels near the eRNA enhancers were higher compared to those in the regions that do not produce eRNA. The distinct overlap observed between EP300, histone modification signals (H3K27ac), and gene expression further validates the accuracy and biological significance of identifying eRNA regions.

**Fig. 3:**
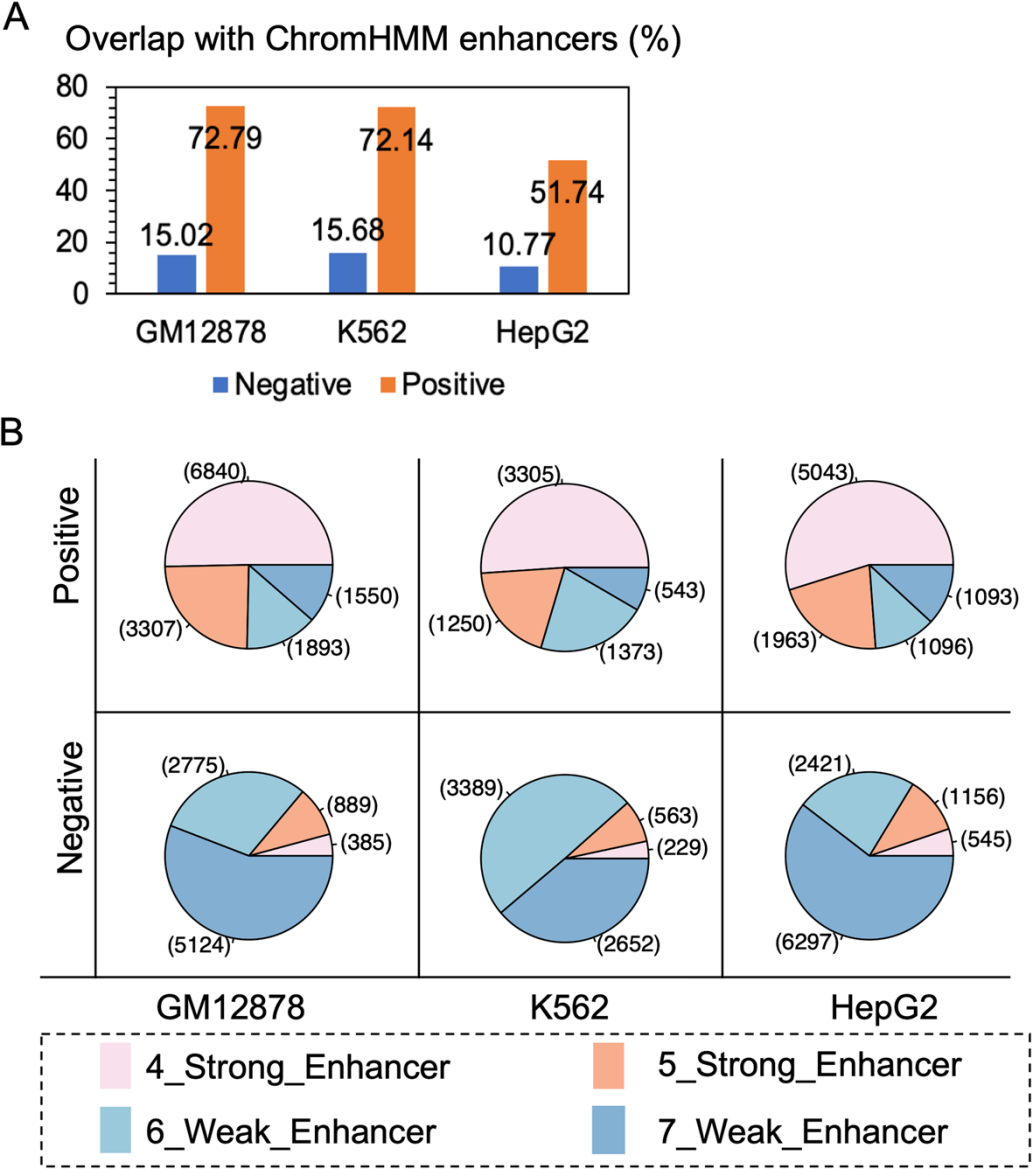
Comparison with ChromHMM. **A** The proportion of regions annotated as enhancers in ChromHMM to positive and negative sample sizes. **B** The distribution of strong/weak enhancers in the region where positive/negative samples overlap with those annotated as enhancers by ChromHMM

In addition, we compared our results with those from ChromHMM annotation. As expected, in the three cells, 72.79%, 71.24%, and 51.74% of the positive examples we identified were annotated as enhancements in ChromHMM (shown in Fig. 3A). Negative examples only accounted for 10.77–15.68%. We conducted further analysis on the overlapping parts. As shown in Fig. 3B most of the samples predicted as positive examples were strong enhancers, while negative examples were mostly weak enhancers. The activity of eRNA and enhancers is related, so it is not unexpected that most positive examples were strong enhancers, while negative samples were mostly weak enhancers. These findings provide strong evidence for the relevance and functionality of the identified eRNAs in the regulatory landscape of the three cell lines. Our research has further confirmed a correlation between the strength of enhancers and eRNA, but the details still need further research.

## Discussion

The biological function of enhancer RNAs has been a subject of debate among researchers, with some considering it to be a result of stochastic transcription. Previous studies often overlooked the noise in CAGE-seq sequencing, which led to false positives in eRNA identification. However, our study successfully mitigates this issue by fitting the distribution of measured eRNA expression levels. By filtering out the noise, it can be inferred that the transcription level of real eRNAs significantly higher than those resulting from stochastic transcription. Reducing the noise interference of stochastic transcription can improve the accuracy of research on the biological function of eRNA and unify scholars' views on the biological function of eRNA. This, in turn, has the potential to contribute to a more unified perspective among scholars regarding the biological functions of eRNAs.

The accuracy of eRNA recognition can be enhanced through the utilization of intra-cell eRNA prediction models. In our model, we incorporated the DNA methylation signal, which provides information about the chromatin state, and the H3K27ac signal as key features. When using DNA methylation alone as the prediction feature, the model achieved an AUC of approximately 0.8. This suggests that additional elements are required to induce eRNA transcription when the chromatin is in an open state. The inclusion of H3K27ac as a feature significantly improved the accuracy of the model, highlighting its importance in facilitating eRNA transcription.

Moreover, the results obtained from cross-model predictions further support the contributions of DNA methylation and H3K27ac to eRNA transcription [29–31]. Our approach to predicting eRNA demonstrates good accuracy, utilizing only two omics datasets. This enhances the accessibility and availability of prediction models for eRNA research. Our prediction strategy can also be extended to identify other regulatory elements. The detection technology for DNA methylation is relatively mature and reliable. The combination of histone modifications and DNA methylation modifications can efficiently reflect the state of chromatin. Integrating histone modifications, DNA methylation modifications, and DNA sequence characteristics can be applied to the recognition of other regulatory elements. In conclusion, our study not only highlights the importance of addressing noise in CAGE-seq expression profiles through a filtering method but also presents an effective eRNA prediction model that relies on a smaller set of omics data types. By reducing the complexity and data requirements, our approach offers a practical and efficient means of studying and predicting eRNA.

## Conclusion

The expression of eRNA detected through CAGE-seq technology is prone to noise due to the inherent variability of RNA expression. To tackle this issue, we employed a filtering approach based on the distribution of eRNA expression obtained from CAGE-seq. This methodology allowed us to filter out noisy signals and get more accurate eRNAs based on statistical methods. However, it is important to note that while this strategy can reduce false positives, it may also increase the likelihood of false negatives.

Furthermore, we have developed a novel method for predicting eRNA by utilizing DNA methylation and histone modification signals. It is worth emphasizing that our prediction models based on traditional machine learning algorithms require limited computational resources. In addition, our models were demonstrated tolerance to the issue of data imbalance in eRNA prediction. The input features utilized in the model are conveniently extracted from biological experiments. Our model is efficient and user-friendly. The R scripts and trained model can be accessed at https://github.com/TracyHIT/eRNA_predict/.

Our experimental findings demonstrate that DNA methylation significantly contributes to the accurate prediction of eRNA, while H3K27ac strongly correlates with enhancer activity and accessible chromatin. By utilizing both DNA methylation and H3K27ac, we can more precisely identify transcriptional enhancers based on the eRNA self-profile obtained through CAGE-seq technology. Moreover, the results of our cross-cell validation indicate that it is feasible to directly predict transcribed enhancers using DNA methylation and H3K27ac. This reinforces the potential of these features as reliable indicators for the identification and characterization of eRNAs across different cell types.

## Methods

### Feature extraction

**Fig. 4:**
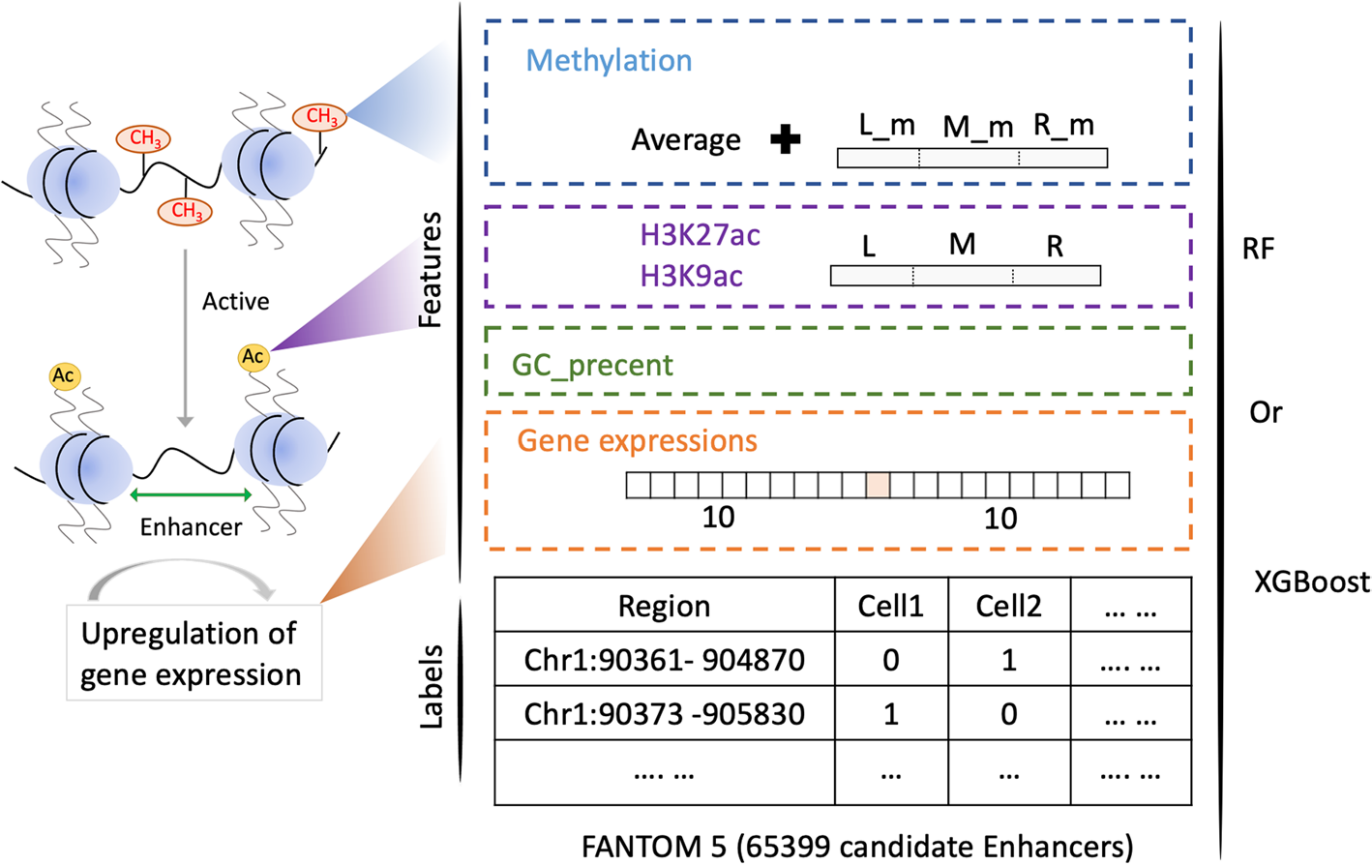
Features and labels used in prediction models

eRNA exhibits several distinct features that can aid in their prediction and identification. These features include: (1) low levels of DNA methylation [30, 32]; (2) specific histone modifications at enhancer loci [31, 33]; (3) accessible (open) chromatin [34]; (4) TF occupancy [35–37]; and (5) RNAP II occupancy [38]. To build a prediction method that is less dependent on omics data, DNA methylation, gene expression, H3K27ac, and H3K9ac were used as input features (as Fig. 4 shows). These data were all downloaded from the ENCODE [39] database. Additional file 2 provide information about these datasets. Generally, the transcription region on the genome is an open and unmethylated region. To capture the DNA methylation characteristics of candidate regions, the average methylation levels in these regions were calculated. Additionally, the candidate region was divided into three segments with an equal length. The average DNA methylation levels of the CpG dinucleotides within each of the three segments were calculated respectively. Totally, these four DNA methylation levels collectively represented the methylation characteristics of the region. Among many histone modification signals, H3 is a marker of the active enhancer. We applied a similar approach to extract histone modification features. It was worth noting that for histone modification signals, we calculate the average coverage of the sequencing reads. eRNA transcription influences the expression of nearby genes bi-directionally. The number of genes regulated by individual enhancers may also vary. The relationship between enhancers and gene regulation is complex. In this study, the expression levels of 10 upstream and downstream genes were also extracted as features. Additionally, the maximum expression level of these 20 genes was also taken into account as gene expression feature. In order to enhance the accuracy of the model without the addition of further biological experimental testing data, sequence features were introduced by counting the frequency of CG content. We then analyzed the importance of a total of 32 features. As shown in Additional file 3, H3K27ac is extremely important in both models. Additionally, the level of DNA methylation and the maximum value of gene expression also possess high predictive value. Due to the limited input features of the model and the need to analyze different experimental data scenarios in our research, we conducted multiple feature combinations. The specific feature combinations are detailed in Table 3.

**Table 3:** Combinations of features were used in prediction models

Index	Feature combination
1	Methyl, GC_precent
2	Methyl, H3K27ac, GC_precent
3	Methyl, H3K9ac, GC_precent
4	Methyl, H3K27ac, H3K9ac, GC_precent
5	Gene_expression, Methyl, H3K27ac, GC_precent
6	Gene_expression, Methyl, H3K9ac, GC_precent
7	Gene_expression, Methyl, H3K27ac, H3K9ac, GC_precent

### Recognition of effective eRNA expression

Our study utilized data from the FANTOM5 database, which includes 65,407 enhancers identified across 1829 cell types detecting by CAGE-seq. The download link is: https://fantom.gsc.riken.jp/5/datafiles/latest/extra/CAGE_peaks/hg19.cage_peak_phase1and2combined_tpm.osc.txt.gz. Due to the extensive coverage of cell types, we proposed that the 65,407 potential enhancers in FANTOM5 likely encompass all regions with the capability of expressing eRNA in human cells. After converting the reference genome from hg19 to GRch38, the dataset contains 65,399 enhancer regions. In the eRNA expression data obtained from CAGE-seq experiments, we observed two distinct data distributions. As an example, we can consider the eRNA data of GM12878. All eRNA expression levels are quantified as TPM. Then, the TPM was logarithmically transformed and linearly amplified using the following formula:

1 $$Lo{g}_{TPM}=10\times \mathrm{ln}(TPM)+4, (TPM>0.001)$$

To better visualize the level of eRNA expression, we converted TPM values to $Lo{g}_{TPM}$. As shown in Fig. 1A, the eRNA expression data exhibits two noticeable distributions. The lower values on the left side of the distribution followed a Poisson distribution. Numerous studies have highlighted the presence of RNA Pol II in a vast number of extragenic regions, emphasizing the prevalence of stochastic transcription events within cells. These lower values likely arise from the noise introduced by the detection technology employed and the inherent stochastic nature of transcriptional noise signals. It is important to note these characteristics when analyzing the eRNA expression data and considering their implications in downstream analyses.

Accordingly, the larger values on the right side of the distribution correspond to effective eRNA expression signals originating from enhancers. Based on the detection technology principle, the eRNA expression distribution follows a negative binomial distribution. We assume that the lower data measurements $x$ from noise follow a Poisson distribution with parameter $u$, while the quantity of eRNA transcribed from enhancers follows a binomial distribution characterized by parameters $r$ and $p$:

2 $${P}_{pois}(x=k)=\frac{{u}^{k}{e}^{-u}}{k!}$$

3 $${P}_{binom}(x=k)=\frac{\left(k+r-1\right)!}{k!\left(r-1\right)!}{p}^{r}{(1-p)}^{k}$$

The final data distribution of fusion is:

4 $$P=a{P}_{pois}(x=k)+(1-a){P}_{binom}(x=k)$$

where $a$ represents the probability of RNA originating from noise transcription. And $1-a$ represents the probability of eRNA transcribed from enhancers. Then maximum likelihood estimators are employed to estimate the parameters of the probability distribution based on the observed data. Nonlinear function optimization is used for maximum likelihood estimation. The interface between two distributions can be considered as a threshold. Values above the threshold suggest a higher likelihood of a true eRNA signal, rather than noise, while values below the threshold indicate the opposite. We then divided the data into two parts. The kurtosis and skewness were calculated for each distribution, and the parameters of the Poisson distribution were estimated for the noise data, resulting in p-values less than 0.05. Therefore, by integrating prior knowledge, the data distributions, and statistic results, we have determined that the two distinct distributions in the CAGE-seq data can be identified.

To enhance the reliability of positive and negative samples during the construction of our algorithm, we included DNaseI data to represent the chromatin state. DNaseI data provide valuable insights into the accessibility of chromatin regions, indicating whether a particular genomic region is open and accessible for transcription factors and other regulatory elements. As shown in Fig. 1B, the regions' $Lo{g}_{TPM}$ values were above the threshold, and the chromatin states of the regions were open, the regions were more likely to be classified as positive samples. Conversely, regions with closed chromatin states and $Lo{g}_{TPM}$ values below the cutoff were considered negative samples. Any regions that do not fall into the categories of positive or negative samples were considered as fuzzy regions, meaning they cannot be clearly defined as either positive or negative examples. Throughout the process of building, training, and evaluating our model, we exclusively utilized the positive and negative samples. These well-defined samples allowed us to effectively train and evaluate the model’s performance, ensuring that it can accurately classify enhancer regions into their respective categories.

### Random forest and XGBoost modeling

In this study, the negative samples outweigh the positive samples, leading to an imbalanced dataset. To tackle this issue, Random forests and the Extreme Gradient Boosting models were employed, as they can tolerate data imbalance. The 65,399 regions were extracted from FANTOM5. According to the expression of CAGE-seq and the coincidence of DNaseI, these regions were divided into three sets: PR (positive regions), FR (fuzzy regions) and NR (negative regions). For the intra-cell analysis, 80% of the regions were allocated for training the model, while the remaining 20% were set aside for independent testing. For the cross-cell analysis, we used data from cells not included in the model training process.

For the random forest model, the number of binary tree variables (mtry) and the number of decision trees (ntree) were two hyperparameters that were fine-tuned by evaluating the out-of-bag error (OOB) one by one. For XGBoost, there were three hyperparameters were considered: nroundsi, max_depthi, etai. They were also selected by OOB. To determine the best values for these hyperparameters, fivefold cross-validation was utilized.

### Model evaluation

To evaluate the performance of models trained using active enhancers and various feature combinations, we utilized the following metrics for evaluation: (1) area under the ROC curve (AUC), (2) sensitivity (Sn), (3) specificity (Sp), (4) Matthew’s correlation coefficient (MCC), (5) F1-score

5 $$Sn=\frac{TP}{TP+FN}$$

6 $$Sp=\frac{TN}{TN+FP}$$

7 $$MCC= \frac{TP\times TN-FP\times FN}{\sqrt{(TP+FP)(TP+FN)(TN+FP)(TN+FN)}}$$

8 $$F1-Score=\frac{2\times TP}{2\times TP+FP+FN}$$

where TP, FP, TN, and FN represent true-positive, false-positive, true-negative, and false-negative values, respectively.

### Supplementary Information

**Additional file 1**. The overlap of positive and negative samples across all three cells. The overlap ratio of positive and negative samples across three types of cells varies greatly. A large number of negative samples are shared among the three types of cells. But positive samples are rarely shared. In three cells, 539 positive regions and 34401 negative regions were shared. Shared negative regions account for 74.66% of all negative regions, while the shared proportion of positive cases is only 4.75%.

**Additional file 2**. The datasets used in the study for extracting input features comes from ENCODE. The files were downloaded from the ENCODE database according to 'EncodeFile_ID'.

**Additional file 3.** The importance of features. The importance of 32 features measured by Gini index in Random Forest model and XGBoost model.

## Data Availability

The datasets analyzed during the current study are available in the FANTOM5 repository, https://slidebase.binf.ku.dk/human_enhancers/. The R script and trained model can be downloaded from https://github.com/TracyHIT/eRNA_predict/.

## Abbreviations

eRNAs: Enhancer RNAs
RF: Random forest
XGBoost: Extreme Gradient Boosting
AUC: Area under the ROC curve
MCC: Matthew’s correlation coefficient
Sn: Sensitivity
Sp: Specificity
TPM: Transcript per million
PR: Positive regions
FR: Fuzzy regions
NR: Negative regions
OOB: Out-of-bag error
