# Supplementary figures and images for: Predicting active enhancers with DNA methylation and histone modification
Source: BMC Bioinformatics. 2023 Nov 2;24:414. doi: 10.1186/s12859-023-05547-y (PMC10621108; doi:10.1186/s12859-023-05547-y)

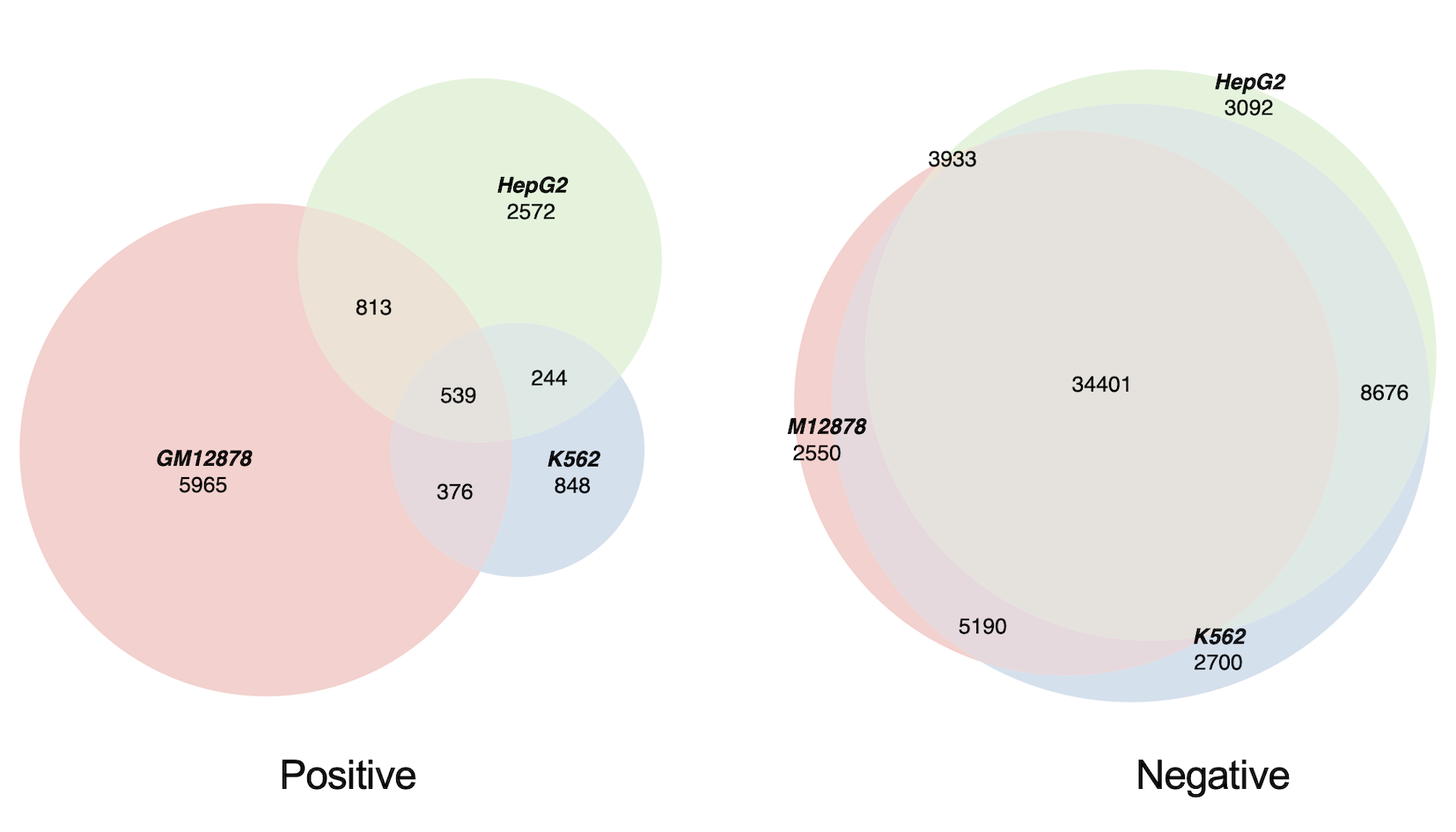

Supplement: Supplementary file 1 — Additional file 1. The overlap of positive and negative samples across all three cells. The overlap ratio of positive and negative samples across three types of cells varies greatly. A large number of negative samples are shared among the three types of cells. But positive samples are rarely shared. In three cells, 539 positive regions and 34401 negative regions were shared. Shared negative regions account for 74.66% of all negative regions, while the shared proportion of positive cases is only 4.75%. [file 12859_2023_5547_MOESM1_ESM.tiff]

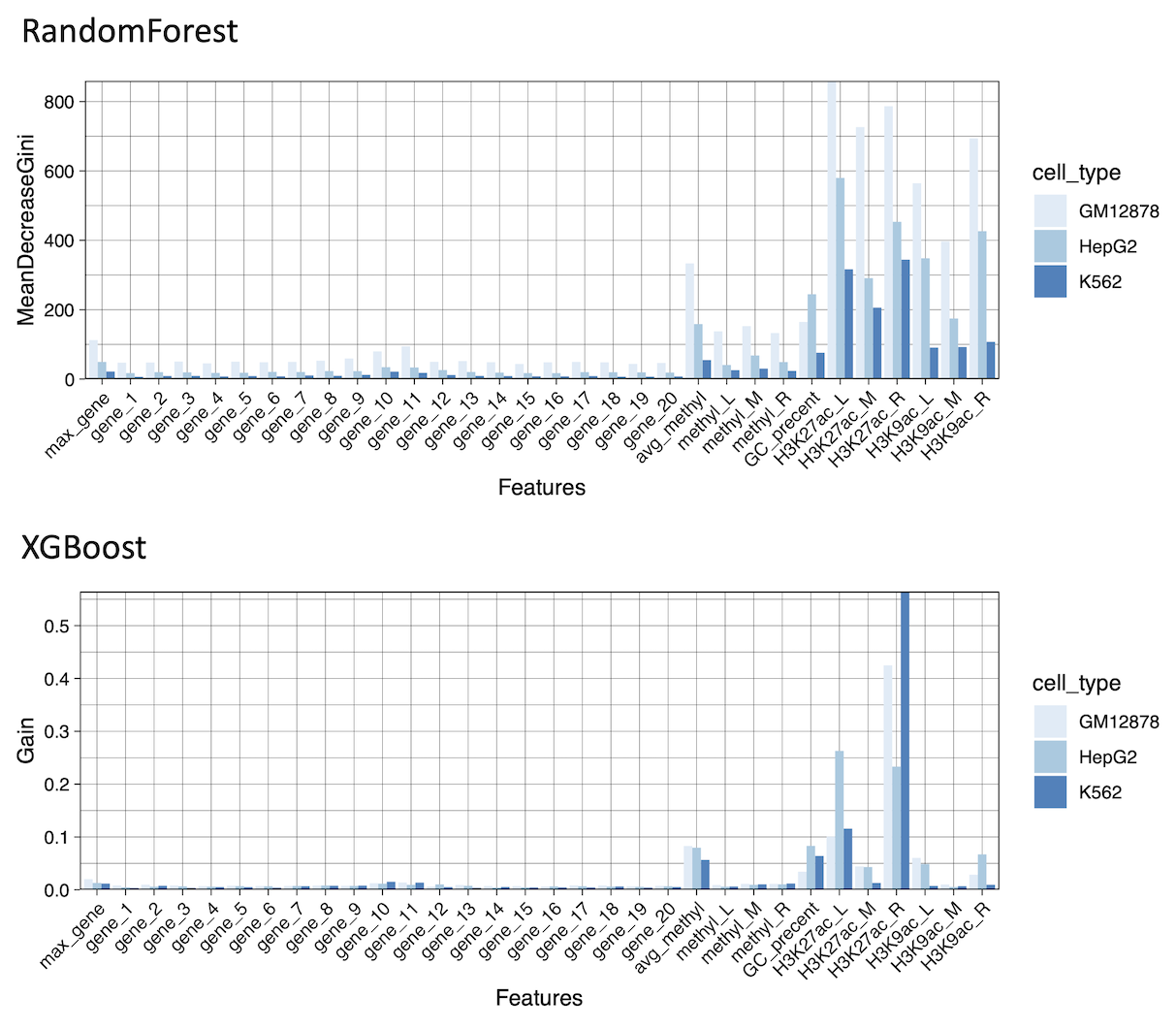

Supplement: Supplementary file 3 — Additional file 3. The importance of features. The importance of 32 features measured by Gini index in Random Forest model and XGBoost model. [file 12859_2023_5547_MOESM3_ESM.tiff]
